# Structural rearrangements in the C-terminal domain homolog of Orange Carotenoid Protein are crucial for carotenoid transfer

**DOI:** 10.1038/s42003-018-0132-5

**Published:** 2018-08-27

**Authors:** Dvir Harris, Adjele Wilson, Fernando Muzzopappa, Nikolai N. Sluchanko, Thomas Friedrich, Eugene G. Maksimov, Diana Kirilovsky, Noam Adir

**Affiliations:** 1 Schulich Faculty of Chemistry, Technion, 3200003 Haifa, Israel; 2 Grand Technion Energy Program (GTEP), Technion, 3200003 Haifa, Israel; 3 Institute for Integrative Biology of the Cell (I2BC), CEA, CNRS, Université Paris-Sud, Université Paris-Saclay, 91198 Gif sur Yvette, France; 4 A.N. Bach Institute of Biochemistry, Federal Research Center, “Fundamentals of Biotechnology” of the Russian Academy of Sciences, Moscow, 119071 Russia; 5 Department of Biophysics, Faculty of Biology, M.V. Lomonosov Moscow State University, Moscow, 119992 Russia; 6 Technical University of Berlin, Institute of Chemistry PC 14, Straße des 17. Juni 135, 10623 Berlin, Germany

## Abstract

A recently reported family of soluble cyanobacterial carotenoproteins, homologs of the C-terminal domain (CTDH) of the photoprotective Orange Carotenoid Protein, is suggested to mediate carotenoid transfer from the thylakoid membrane to the Helical Carotenoid Proteins, which are paralogs of the N-terminal domain of the OCP. Here we present the three-dimensional structure of a carotenoid-free CTDH variant from *Anabaena* (*Nostoc*) PCC 7120. This CTDH contains a cysteine residue at position 103. Two dimer-forming interfaces were identified, one stabilized by a disulfide bond between monomers and the second between each monomer’s β-sheets, both compatible with small-angle X-ray scattering data and likely representing intermediates of carotenoid transfer processes. The crystal structure revealed a major positional change of the C-terminal tail. Further mutational analysis revealed the importance of the C-terminal tail in both carotenoid uptake and delivery. These results have allowed us to suggest a detailed model for carotenoid transfer via these soluble proteins.

## Abstract (web summary)

Dvir Harris et al. present the structure of a homolog of the orange carotenoid protein (OCP) C-terminal domain, elaborating on this protein family’s carotenoid transfer mechanism. They observed major structural shifts in the homolog compared to that of the OCP C-terminal domain, with a strong positive impact on carotenoid uptake and delivery.

## Introduction

Photosynthetic organisms, including cyanobacteria, utilize the energy of the sun to sustain themselves in a wide range of static and dynamically changing conditions. The photosynthetic process is initiated by light absorption by antenna complexes, which are the phycobilisomes^1,2^ in cyanobacteria. Excess absorbed energy can become deleterious without proper regulation as overexcitation may cause damage to the photochemical reaction centers, leading to cell stress and eventually cell death^3^. Cyanobacteria have evolved several protection mechanisms including non-photochemical quenching, induced by the Orange Carotenoid Protein (OCP)^4,5^. OCP is a water-soluble, carotenoid-binding, 35 kDa protein found to induce dissipation of phycobilisome excitation energy minimizing energy flux toward the reaction centers^5–7^. OCP can also directly protect against the presence of singlet oxygen^8,9^. OCP is comprised of two domains: the N-terminal domain (NTD) and the C-terminal domain (CTD) connected through an inter-domain flexible linker loop^9^. In darkness and low light, OCP is in its stable inactive orange state (OCP^O^). Upon strong illumination, changes in the carotenoid and the protein lead to the formation of the metastable, active red state (OCP^R^) in which CTD and NTD are completely separated^6^. Only the OCP^R^ state interacts with the phycobilisome to induce fluorescence quenching through its NTD^10,11^. The CTD also interacts with the fluorescence recovery protein, which supports the reorganization of OCP^R^ to OCP^O^^12–14^. The structure of OCP^O^ (PDB code: 5UI2, Supplementary Figure 1) demonstrated that the carotenoid is situated in a hydrophobic cavity formed by both the CTD and the NTD^9,15–18^.

Although the structure of the OCP^R^ state remains elusive to date, the structure of the isolated NTD with bound carotenoid (holo-NTD) was determined (PDB code: 4XB4)^16^. This holo-NTD structure revealed that, upon photoactivation, the carotenoid becomes more planar and is translocated by 12 Å into the NTD cavity^16^. The crystal structure of a carotenoid-free CTD (apo-CTD) has not yet been determined (Supplementary Figure 1). Phylogenetic studies discovered that cyanobacteria contain genes that encode for homologs of both the NTD and CTD^19–21^. NTD homologs have been termed Helical Carotenoid Proteins (HCPs) for their all α-helical structure^19,22^. *Anabaena* (*Nostoc*) PCC 7120 (hereafter *Anabaena*) possesses four variants of HCPs (out of nine total clades identified in cyanobacteria^19^). The holo-HCP1 was isolated from *Anabaena* and its structure was solved, proving HCPs to be carotenoid-binding proteins^19^. Its structure exhibited high similarity to the NTD of OCP, which can individually exist as a proteolytic cleavage product of OCP^10^. Previous studies have shown that each of the HCP clades demonstrates different features^22^. Unlike the wide variety of HCPs, the CTD homologs (CTDH) fall only into two clades, of which only one is present in *Anabaena* (a clade 2 CTDH). The CTDH gene in *Anabaena* is located adjacent to the HCP4 gene, therefore it was suggested that HCP4 and CTDH are the progenitors of OCP^19,23,24^.

Recently, it was revealed that isolated CTD of *Synechocystis* OCP and CTDHs from *Anabaena* and *Thermosynechococcus elongatus* (a clade 1 CTDH) are also capable of carotenoid binding^25,26^. Both CTD and CTDH were shown to mediate carotenoid transfer to apo-OCP, apo-NTD, or apo-HCP (from clade 4)^25,26^. OCP and CTDH are also capable of taking up a carotenoid molecule from membranes while HCP4 and isolated NTD are unable to do so^25^. Thus one likely role of CTDHs is to serve as intermediate transfer molecules required for delivery of carotenoids from membranes to the HCPs. *Anabaena* CTDH was shown to form a disulfide-linked dimer in the presence or absence of carotenoid under oxidizing conditions. By contrast, the CTDH from *T. elongatus* (hereafter TeCTDH) that lacks Cys103 forms a dimer only in the presence of a carotenoid that is shared by the two monomers^25^. When stabilized by the presence of the disulfide bond, the CTDH dimer could not donate its carotenoid to an acceptor molecule; the reduced form of holo-AnaCTDH was able to transfer its carotenoid to HCP. This suggests that the disulfide bond containing clade 2 CTDH dimer has additional regulation and may be controlled by changes in the redox state in the cell, functioning upon stress induction.

We present here three important findings on the carotenoid transfer mechanism by CTDH. First, there is a major structural shift of the C-terminal tail of the CTDH (with respect to its position in the holo-form of CTD-OCP), bringing it into close proximity to the carotenoid-binding pocket. Second, we show that the C-terminal tail has a strong positive impact on both the carotenoid uptake as well as the carotenoid delivery by the CTDH; when absent, the rates of these processes are considerably reduced. Finally, the role of CTDH as carotenoid donor to HCPs was confirmed. Surprisingly, apo-AnaCTDH was shown to be also capable of receiving a carotenoid molecule from HCP1, suggesting an increased complexity for carotenoid transfer between different partners.

## Results

### The AnaCTDH can form a large homogeneous oligomer

*Anabaena* apo-CTDH was prepared for crystallization as described in Muzzopappa et al. (2017)^25^. Native-polyacrylamide gel electrophoresis (native-PAGE) indicated that the major assembly is a dimeric form, suggesting the presence of a disulfide bond between the CTDH monomers (Supplementary Figure 2A). Under reducing conditions, the CTDH appeared as a monomer (Supplementary Figure 2B). Size exclusion chromatography revealed that the CTDH protein in solution was mostly in a higher oligomeric state (Supplementary Figure 3, dashed bold line) with additional, smaller oligomeric states in solution. This discrepancy concerning AnaCTDH oligomeric state between native-PAGE and size exclusion chromatography has previously been published^25^.

Crystallization trials were attempted on this apparent mixture of forms and large crystals were obtained (Supplementary Figure 4A). Using a laboratory X-ray source, the crystals diffracted to 2.9 Å (Supplementary Figure 4B). Indexing revealed a rather large unit cell (Table 1) with respect to the molecular weight (MW) of the AnaCTDH dimer (34 kDa). The calculated Matthews coefficient indicated that the asymmetric unit could contain 10–24 AnaCTDH monomers.

**Table 1:** Data-collection and final refinement statistics

	Native	2 M urea
Data collection		
Space group	*P*2_1_2_1_2_1_	*P*6_5_22
Cell dimensions		
*a*, *b*, *c* (Å)	65.65, 112.28, 318.54	81.08, 81.08, 162.94
*α*, *β*, *γ* (°)	90.0, 90.0, 90.0	90.0, 90.0, 120.0
Resolution (Å)	47.99–2.90 (3.06–2.90)^a^	53.19–2.75 (2.85–2.75)^a^
* R*_merge_	0.06 (0.42)^a^	0.07 (0.33)^a^
〈*I*/*σ*(*I*)〉	16.7 (3.3)^a^	11.7 (5.0)^a^
Completeness (%)	99.9 (100)^a^	99.8 (100)^a^
Redundancy	3.7 (3.9)^a^	5.6 (6.1)^a^
Refinement		
Resolution (Å)		53.19–2.75
No. of reflections		8742
* R*_work_/*R*_free_		0.241/0.292
Completeness (%)		99.58
No. of non-hydrogen atoms		1855
Protein		1828
Ligands		12
Water		15
B factors (Å^2^)		
Protein		67.31
Ligands		78.36
Water		45.16
R.m.s. deviations		
Bond lengths (Å)		0.012
Bond angles (°)		1.47

Values in parentheses are for the outer shell

Whether this large oligomerization state has any biological significance as a quaternary assembly has to be further investigated. The fact that it was successfully crystallized and diffracted well suggests that this state is not a random aggregation and that different faces of interaction between the monomers (or between dimers) must exist. The presence of high MW oligomers was also detected in carotenoid-containing versions of AnaCTDH, TeCTDH, and *Synechocystis* CTD-OCP preparations obtained in *Escherichia coli* cells^25^, suggesting that oligomerization is a more general characteristic of CTD-like proteins. Nevertheless, these large oligomers were not detected in size exclusion chromatography when the holo-proteins were isolated from *Synechocystis* cells or when AnaCTDH was isolated under reducing conditions or in a AnaCTDH Cys103Phe mutant^25^.

### Crystallization of apo-AnaCTDH dimer

In order to obtain a more homogeneous preparation of apo-AnaCTDH dimers, we added 2 M urea to the solution^27,28^, which caused a large proportion of the high oligomeric states to disassemble, resulting in predominantly dimeric CTDH (Supplementary Figure 3, solid line). Crystals (Supplementary Figure 5A) were obtained in the presence of urea (see Methods). The apo-AnaCTDH dimer crystals were found to diffract to 2.43 Å with synchrotron radiation (Supplementary Figure 5B, and Table 1).

### Determination of the urea-treated dimeric apo-AnaCTDH structure

**Fig. 1:**
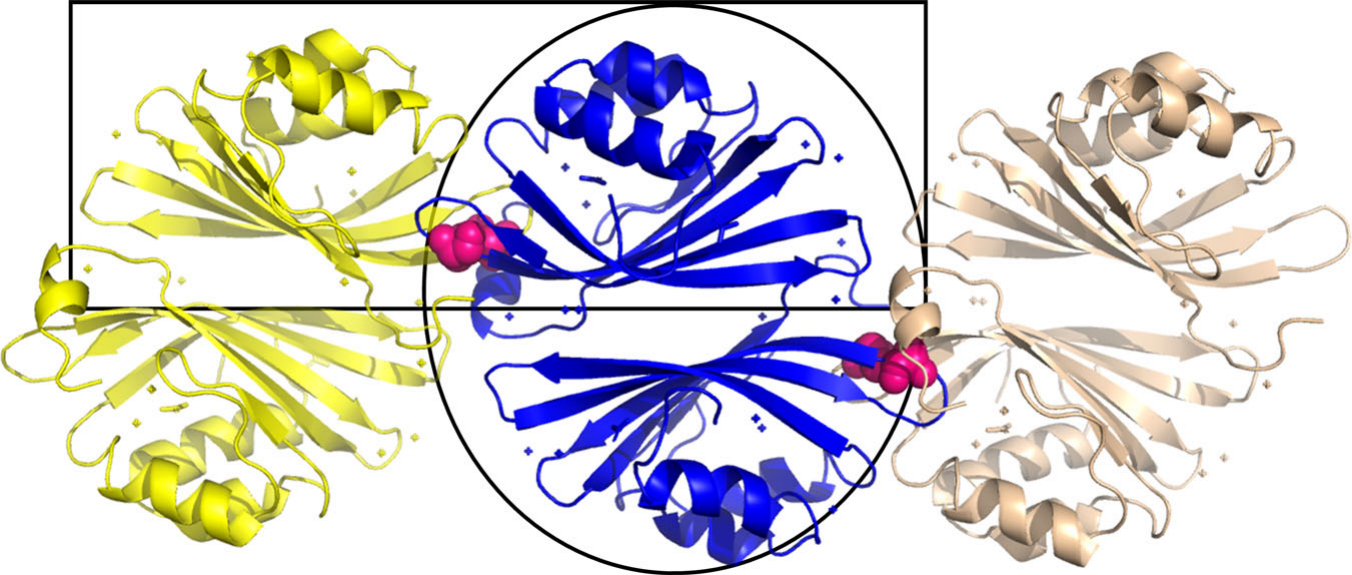
Two dimeric interfaces are revealed in the CTDH structure. Three adjacent asymmetric units are depicted in yellow, blue, and light pink. The Cys103 residues forming the disulfide bonds are presented as pink spheres. The asymmetric unit contains a back-to-back dimer interface (called A-type; black oval). The disulfide bonds link the head-to-head functional dimer (F-type; black rectangle)

The X-ray crystallographic structure of apo-AnaCTDH was determined by molecular replacement (MR) to 2.75 Å resolution using the OCP^O^-CTD as the search model (from the 5UI2 structure), with two monomers in the asymmetric unit. Somewhat surprisingly, the disulfide bond between Cys103 residues of two monomers mentioned above was found to be between two different asymmetric units and not between the two monomers of one asymmetric unit. The asymmetric unit dimer (hence called A-type or back-to-back) was formed via an interaction plane between the β-sheets of two monomers, quite distant from Cys103 (Fig. 1). The presence of the oxidized disulfide bond was confirmed by the presence of continuous electron density between the two Cys103 residues in the Fo-Fc omit map (Supplementary Figure 6).

Analysis using the PISA macromolecular interaction tool^29^ showed that both the A-type dimer and the functional disulfide-containing dimer (hence called an F-type or head-to-head) exhibited negative calculated Δ*G* values, predicted to be stable. PISA suggested that only the F-type dimer has biological significance, (Δ^i^*G*_*P* value_ of 0.067 for the F-type dimer interface). This finding agrees with the suggestion proposed by Moldenhauer et al. (2017)^26^ and Muzzopappa et al. (2017)^25^ that the interaction plane between the CTD(H) dimer should be at the same site as the CTD and NTD in OCP^O^. In contrast, a Δ^i^*G*_*P* value_ of 0.518 in the case of the A-type dimer interface implies possibly crystal-stabilized interface (as a Δ^i^*G*_*P* value_ <0.5 is considered necessary for biological significance^29^). However, this interface might still be important for the assembly into large oligomers in solution, which was shown to be condition-dependent^25^ as well as a possible different type of dimeric interaction under some specific conditions.

### Analysis of the dimeric interfaces in solution

The presence of the disulfide bond forces the formation of the F-type dimer of apo-AnaCTDH. However, upon the reduction of cysteines or in their absence (in clade 1 CTDHs or in CTD-OCP), the A-type dimerization can become possible. The heterogeneous nature of the apo-AnaCTDH solution (Supplementary Figure 3) and this protein’s propensity to monomerize in the absence of carotenoid^25^ impedes structural analysis of its non-covalently bound dimers. In contrast, individual apo-CTDs from *Synechocystis* OCP (carrying a Phe instead of Cys at residue position 103) efficiently dimerize without forming higher-order oligomers^26^, making apo-CTD dimers suitable for structural characterization in solution by using small-angle X-ray scattering (SAXS).

**Fig. 2:**
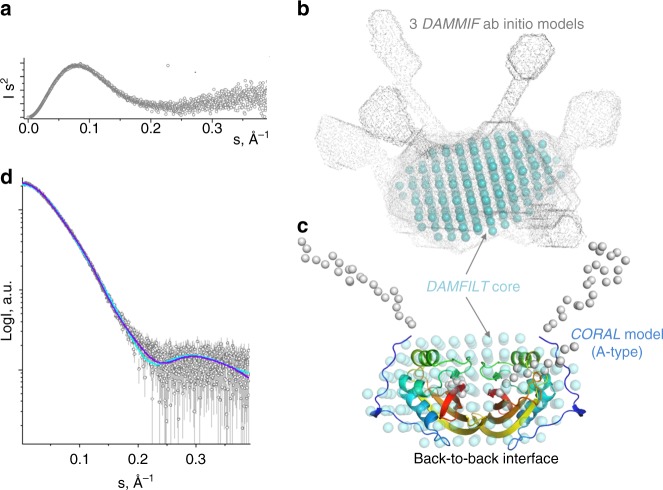
Analysis of the solution conformation of Synechocystis apo-CTD dimers using SAXS. **a** Kratky plot showing that apo-CTD is represented by particles with a globular core and flexible termini/loops. **b** Ab initio shape reconstruction using *DAMMIF*. Three best-fitting *DAMMIF* models are shown superimposed to reveal the common core (*DAMFILT* core, cyan spheres). **c** *CORAL*-derived structural model of the A-type apo-CTD dimer obtained upon modeling of the flexible N- (23 residues) and C-terminal (12 residues) tails in order to minimize the discrepancy between the experimental and theoretical SAXS curves calculated from the model. The *CORAL* model overlaid with the *DAMFILT* core (cyan spheres) from **b** is shown by ribbons colored by gradient from blue (N-terminus) to red (C-terminus), with the flexible termini represented by Cα atoms (gray spheres). **d** Fitting of the experimental SAXS profile for apo-CTD by the best-fitting *CORAL*-derived models corresponding to either A-type (dark blue; *χ*^2^ = 1.14, CorMap = 0.16) or F-type (light blue; *χ*^2^ = 2.51, CorMap = 0.00) dimers

At 270 µM, the 18.7 kDa apo-CTD construct gave particles with a radius of gyration (*R*_g_) of 2.7 nm and the maximum particle dimension (*D*_max_) of 9.5 nm. The bell-shaped Kratky plot with the gradual rise of the curve at high angles (Fig. 2a) suggested particles with a compact globular core and moderate flexibility. The Porod volume of 56 nm^3^ suggests that the corresponding MW estimate (35 kDa) is close to that expected for apo-CTD dimers (37 kDa), in line with their known dimerization at concentrations above 150 µM^26,30^. Ab initio molecular shape reconstruction using *DAMMIF*^31^ resulted in globular models with pairs of protrusions at variable positions (Fig. 2b), consistent with the Kratky plot. Averaging of the *DAMMIF* models with *DAMAVER*^32^ revealed a core, common for the generated models (*DAMFILT* core; shown by cyan spheres in Fig. 2b). Given the similarity of the primary structure and fold of apo-CTD from *Synechocystis* and apo-AnaCTDH (Cα r.m.s.d. <1 Å), the two types of dimeric cores found in the crystal lattice (Fig. 1) were built by spatial overlay, yielding either the F-type or the A-type dimer. To account for local differences between the apo-CTD and apo-AnaCTDH structures, the A-type dimer of apo-CTD was further refined by local protein–protein docking approach using *RosettaDock* server^33^, which resulted in a more connected, realistic structural model free from steric clashes (Supplementary Figure 7A). This model, matching the *DAMFILT*-derived globular core (Fig. 2c), was used as a template to model the unstructured terminal regions (including the C-terminal tails) using *CORAL* (Fig. 2c). Likewise, the F-type of the apo-CTD dimer was slightly corrected to eliminate apparent clashes and then used to build a *CORAL* model with flexible termini. *CORAL*-derived structural models for both types of dimers described the experimental SAXS data reasonably well; however, statistical analysis including χ^2^ and the correlation map *P* value (CorMap)^34^ showed much better fits in the case of A-type dimer (Fig. 2d). This favored fit for the A-type apo-CTD dimer may indicate that, in the absence of the Cys–Cys bond, the back-to-back interface might be more favorable in solution. It is likely that the compromised ability of apo-AnaCTDH to form non-covalent dimers, which presumably occurs only at high protein concentrations, is associated with the absence of the functionally important phenylalanine residues in the external side of the β-sheet (in positions 299 and 300 of *Synechocystis* OCP), which in AnaCTDH are occupied by Ile-His residues (see Supplementary Figures 7B and 7C). This hypothesis is in line with the reported inability of apo-TeCTDH lacking a Phe in position homologous to 299 in the CTD of *Synechocystis* OCP to form stable dimers^25^. Attempts to fit the SAXS data with either a monomer or a trimer of apo-CTD gave significantly poorer fits (*χ*^2^ ~ 19 for a monomer, *χ*^2^ ~ 5 for a trimer).

These observations indicate that transitions between the two types of dimers may occur, which may be relevant for the carotenoid transfer mechanism. Owing to the steric constraints, the F-type crystallographic apo-AnaCTDH dimer is not capable of performing carotenoid uptake from the membranes, as the carotenoid channel is partially blocked by the C-terminal tail and filled with the Cys103-containing loop (hereafter β5/β6 loop), while in the A-type dimer, the carotenoid cavity is open. Notably, the carotenoid transfer is suppressed when the dimeric holoform is stabilized by the disulfide bond^25^. Furthermore, the A-type conformation may represent one of the intermediates during carotenoid transfer into the HCPs. One may speculate that, during the transfer, the F-type dimer disassembles, exposing one side of the carotenoid, and yields an intermediate A-type dimer with the carotenoid accessible in one holo-monomer, which then forms a new F-type-like interface with an apo-HCP counterpart.

### Structural changes related to the shortening of CTDH relative to CTD

**Fig. 3:**
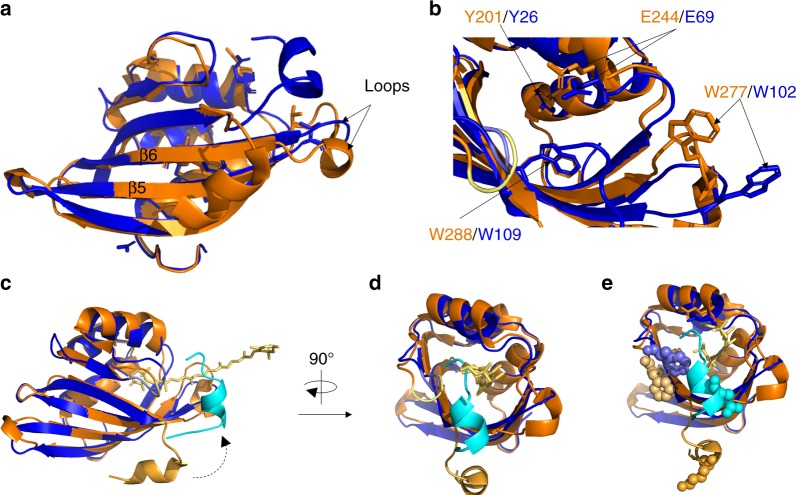
Crystal structure comparison between apo-AnaCTDH and CTD OCP^O^ (PDB code: 5UI2). **a** CTD-OCP (PDB code: 5UI2, orange) and apo-AnaCTDH (blue) structures are superimposed. Both elongation of β6 strand and shortening of the loop connecting β5 and β6 strands are observed for apo-AnaCTDH. **b** These differences lead to spatial changes in both E244/E69 and W277/W102. In contrast, Y201/Y26 and W288/W109 overlap well in both structures. **c** Cartoon representation of CTDH (blue shades) super-positioned with CTD of OCP^O^ (orange shades). The C-terminal tail changes its orientation (indicated by dashed arrow) substantially from an external position (tail in orange) to an internal position for apo-AnaCTDH (tail in light blue). The position of the carotenoid molecule (yellow stick representation) is preserved from the 5UI2 structure. **d** Same as **c** but rotated clockwise by 90°. **e** Same as **b** but with amino acids known to be substantially perturbed in their solvent accessibility upon holo-to-apo transition in CTD-OCP

To date, the only high-resolution structural information available on the CTD is when it is in association with the NTD in the OCP^O^ state of the full-length OCP protein (closed carotenoid-associated conformation–PDB codes: 5UI2, 4XB5, 5TUX, 3MG1, etc.). As opposed to the CTDs of OCPs, all CTDHs lack the four amino acids that immediately follow residue 103 (residue 278 in *Synechocystis* OCP) in the CTD (Supplementary Figure 7C). Therefore, tertiary structure differences were expected to be present between the proteins^25^. Homology modeling suggested that, in the CTDH, the gap would result in a shortening of the β6 strand, while the loop that connects this strand with β5 would become longer forcing it into a different position^25^. The resolution of the three-dimensional structure of apo-AnaCTDH presented in this study shows that the lack of these four amino acids causes structural changes, different than those predicted by modeling. The β6 strand is longer and the loop between strands β6 and β5 is shorter (Fig. 3a). These structural changes lead to a change in the position of Glu244 and Trp277 (Fig. 3b), which play an essential role in the interactions between the CTD and NTD in the OCP^O^ state^15^. These changes in positions can affect assembly processes, which in turn result in diverse activities by different protein pairs. More specifically, when holo-CTD and apo-NTD interact, an OCP-like protein is formed, in which the carotenoid is shared by the CTD and NTD (similarly to OCP^O^); however, when holo-CTDH and apo-HCP interact, the carotenoid is not shared by both domains since full carotenoid transfer occurs (from CTDH to HCP)^25^. In contrast to the residues noted above, Tyr201 and Trp288 in *Synechocystis* CTD (Tyr26 and Trp109 in *Ana*CTDH), which are found in interaction with the carotenoid in OCP^O^, are in almost identical positions in OCP and in the apo-AnaCTDH structure presented here, suggesting that the carotenoid can interact with these amino acids in CTDH dimers.

In an engineered AnaCTDH mutant in which the four amino acids missing in the sequence (Supplementary Figure 7C) were reintroduced and the Cys103 was changed to Phe, the holo-dimer was less stable and easily lost the carotenoid^25^. This suggests that the structural change induced by the lack of the four amino acids is important for carotenoid stabilization in the CTDH dimer. Here we show that the addition of the 4 amino acids also hinders carotenoid uptake. Holo-HCP4, -HCP3, and -HCP2 were unable to give the carotenoid to apo-AnaCTDH-C103F mutant protein (which cannot form the critical disulfide bond, thus making carotenoid transfer possible) (Supplementary Figure 8A, 8B, and 8C).

**Fig. 4:**
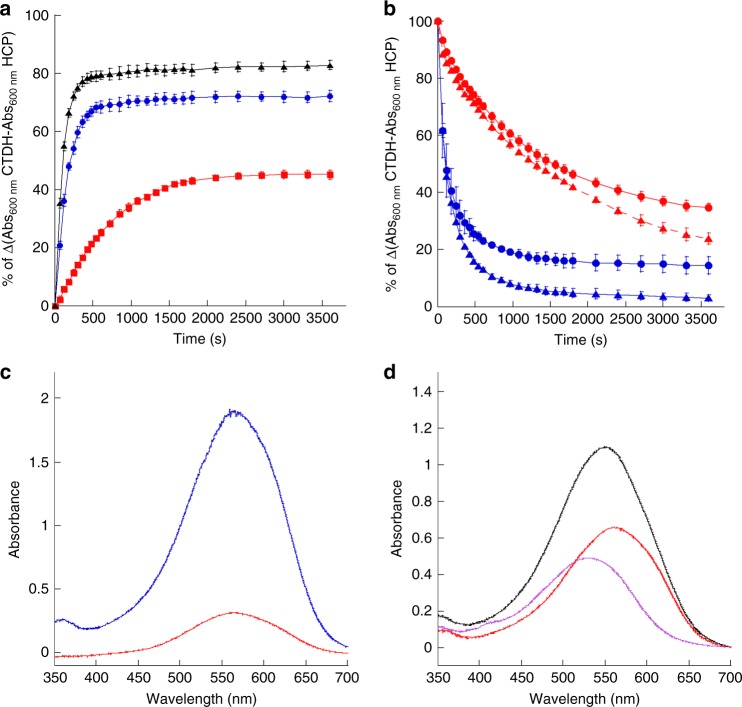
Carotenoid transfer between HCPs and CTDHs and carotenoid uptake from membranes. **a** Kinetics of carotenoid transfer from holo-HCP1 to apo-CTDH-C103F-Cter-Histag (blue), apo-CTDH-C103F-Nter-Histag (black), and apo-CTDH-C103F+4aa (red) followed by an increase of absorbance at 600 nm. **b** Kinetics of carotenoid transfer from holo-CTDH-C103F (blue) and from holo-CTDH-C103F-∆C-terminal tail (∆C-terminal tail abbreviated to CTT on spectra, red) to apo-HCP1 (triangles) and apo-HCP4 (circles) followed by the decrease of absorbance at 600 nm. The CTDHs used in these experiments have the His-tag in the C-terminus. **c** Absorption spectra of the supernatant after 1 h incubation of canthaxanthin containing *E. coli* membranes with apo-CTDH-C103F (blue) and Apo-CTDH-C103-∆C-terminal tail (red) following membranes removal. 100% of apo-CTDH-C103F was converted to holo-protein while only 20(±5)% of apo-CTDHC103F-ΔC was converted to holo-protein. **d** Absorption spectrum of the supernatant after 1 h incubation of canthaxanthin containing *E. coli* membranes with apo-CTDH-C103F and apo-HCP1 (black). The membranes were removed before spectrum recording. Deconvolution of the graph into HCP1 (46%) and CTDH (54%) components is also shown. The ratio CTDH to HCP1 was 1 during the incubation. The experiments were done at least three times and protein concentrations were similar

Surprisingly, HCP1 largely transferred its carotenoid to apo-AnaCTDH-C103F (Fig. 4a, blue and black spectra, and Supplementary Figure 9A). The carotenoid transfer was slower from HCP1 to apo-AnaCTDH-C103F+4aa (Fig. 4a, red spectrum), indicating that this CTDH mutant is hindered in the uptake and stabilization of the carotenoid. In conclusion, in CTDHs, the structural changes generated by the absence of the four amino acids following Cys103 are critical for carotenoid uptake, binding, and stabilization of the dimer.

### The position of the C-terminal tail in the CTDH structure

The most striking difference between the apo-AnaCTDH structure presented here and the CTD-OCP^O^ structure is a major shift in the position of the C-terminal tail (Fig. 3c). In the CTD-OCP^O^ structure, the C-terminal tail is in an external position interacting with the β-sheets and possibly with the N-terminal extension (based on their proximity, as demonstrated in 5UI2 structure). In contrast, in the apo-AnaCTDH structure, the C-terminal tail adopts an internal position, coming closer to the F-type interface between the CTDH monomers (CTD-NTD interface in OCP), partially covering the opening of the designated carotenoid-accommodating cavity in the apo-AnaCTDH structure (Fig. 3d).

This dramatic shift in the position of the C-terminal tail is in good agreement with the literature—the structural changes taking place upon OCP^O^ to OCP^R^ photoconversion were studied previously^17^ by examining amino acid solvent accessibility. It was demonstrated that residues Pro225-Pro226-Phe227 (Phe227 is shown as shaded wheat-orange spheres in Fig. 3e) exhibit the second highest decrease in accessibility in the CTD upon phototransformation. It was postulated that this PPF patch serves as a cap for the carotenoid cavity in the CTD upon translocation of the carotenoid in the OCP^R^ state. However, Lys310 (bottom-most light orange spheres in Fig. 3e) exhibited the highest decrease in accessibility, yet no explanation was suggested for this observation. The apo-AnaCTDH structure provides a more comprehensive interpretation of these results. Upon loss of the carotenoid to the NTD, the C-terminal tail, which points away from the OCP^O^ globular structure and was highly accessible, now rotates by nearly 180° and turns into the carotenoid cavity, capping it. This capping function covers the PPF patch completely while internalizing Gln132, the structurally homologous CTDH residue of Lys310 in the CTD (Fig. 3e).

### Role of the C-terminal tail

In order to assess the role of the C-terminal tail, two AnaCTDH-C103F mutants lacking the C-terminal tail were constructed. Both mutated proteins lacked 11 C-terminal amino acids from Leu126 to Leu136. One of the proteins had the His_6_-tag at the C-terminus just after Lys125 while the second mutant had the His_6_-tag at the N-terminus (see Methods). Regardless of the position of the His-tag, the mutated CTDH-C103F-ΔC-terminal tail failed to receive a carotenoid from holo-HCP1 (Supplementary Figures 8E and 8F). The carotenoid uptake from the membranes was also largely reduced when the C-terminal tail was absent (only 20% of holo-protein was produced (Fig. 4c).

The importance of the C-terminal tail was further examined for the carotenoid delivery by the holo-CTDH. For both apo-HCP1 and apo-HCP4 as recipients, holo-CTDH was able to transfer its carotenoid with or without the C-terminal tail (Supplementary Figures 9b and 9c), yet the absence of the C-terminal tail from the holo-CTDH decreased the rate of carotenoid transfer (Fig. 4b).

These results demonstrate that the internal, cavity-blocking position of the C-terminal tail in the apo-form does not hinder carotenoid uptake. In fact, it facilitates its uptake from both the membrane and, surprisingly, from holo-HCP1. Moreover, the C-terminal tail facilitates transfer the carotenoid to HCPs, possibly starting from its external position (as in OCP^O^), then moving closer to the carotenoid to expedite the process. Therefore, the C-terminal tail, which starts with a relatively hydrophobic patch and ends in a relatively hydrophilic patch (Supplementary Figure 7c), may serve as a facilitator for carotenoid uptake and delivery. For uptake, the hydrophilic end may bind to the hydrophilic lipid head groups followed by forming an interaction between the hydrophobic patch and the membrane-embedded carotenoid molecule. For delivery, the hydrophilic patch would bind to the appropriate site on the HCP, followed by transfer of the carotenoid to the HCP-binding cavity, facilitated by the hydrophobic patch.

### Possible structural changes anticipated in CTDH following carotenoid uptake

The apo-CTDH structure is expected to change upon carotenoid binding, so we attempted to understand the structural effects of canthaxanthin binding on apo-AnaCTDH. Computational docking of a carotenoid molecule to the apo-AnaCTDH structure was executed using the Swissdock server^35^. When the F-type dimer was used as the host (with or without the C-terminal tails), not a single 1 of the 34 docking clusters predicted a carotenoid positioned inside the cavity formed by the dimer (Supplementary Figures 10A and 10B). Manual positioning of the carotenoid molecule was also unsuccessful. Thus the F-type dimer cannot accommodate the carotenoid unless structural changes occur. Indeed, size exclusion chromatography and native-PAGE showed different volumes for holo- and apo-AnaCTDH^25^. The same situation seems to be valid for CTD-OCP, where the holo-dimer was reported to have a larger apparent size than the apo-dimer^26^.

By contrast, when an apo-AnaCTDH monomer was used as the host (with or without the C-terminal tail), 31 out of the top 34 clusters suggested that the carotenoid would be positioned in the pocket that has been shown to bind the carotenoid in OCP^O^ (Supplementary Figures 10C and 10D). In both docking trials, the outward-facing region of the C-terminal tail was found to be a good docking site for the carotenoid molecule (red circles, Supplementary Figures 10B and 10D), suggesting that there could be an interaction between the two (as proposed above).

Two possible structural obstacles for carotenoid accommodation in the F-type CTDH dimer based on the apo-AnaCTDH structure can be suggested. First, the β5/β6 loops (Gln100-Gly106, see Supplementary Figure 11), from both monomers, fill the opposing cavities (Supplementary Figure 11), which together are suspected to host the carotenoid molecule in the holo-AnaCTDH dimer form as inferred from the OCP^O^ structure. When using an apo-AnaCTDH monomer as a docking target, the β5/β6 loop from the other monomer is absent in the cavity, thus possibly enabling the carotenoid molecule to be docked in the monomeric target. Based on the scenario described above, we suggest that, upon carotenoid association, the position of these loops is altered, facilitating the binding of the carotenoid.

In addition, as already noted, the volume of holo-AnaCTDH is larger than that of apo-AnaCTDH. This could be related to a slight separation of monomers to yield a sufficiently sized cavity, long enough to accommodate the carotenoid. The distances between Trp110–Trp110’ and Tyr27–Tyr27’ in the F-type dimer (~25 and ~30 Å, respectively) are too small for the accommodation of a canthaxanthin molecule (~30 Å), especially if these amino acids should be allowed to form H-bonds with the carotenoid carbonyls. The Trp288Ala mutant of CTD-OCP was shown to transfer the carotenoid more easily than the wild type (WT)^30^. This implied that the Trp stabilizes the binding of the carotenoid in CTD(H)s as in full-size OCPs.

It is important to note that carotenoid-containing monomeric forms of CTD(H)s were detected previously^25,30^. We thus hypothesized that a monomer can temporarily accommodate the carotenoid taken up from a membrane. Carotenoid binding could occur during CTDH synthesis, although uptake into apo-AnaCTDH has been shown experimentally^25^. The question arises about how this is possible with the position of the C-terminal tail in apo-AnaCTDH acting as an intermediate cap of the carotenoid-binding site. As discussed, we observed that not only apo-AnaCTDH can take the carotenoid from the membrane but also from HCP1. These results suggest that the C-terminal tail moves rather easily. Moreover, our results indicate that the C-terminal tail facilitates carotenoid uptake and delivery. It was suggested that the position of the carotenoid in the monomer (completely buried) must be different to that in the dimer in which it is shared by two monomers^25,30^. We propose that, first, the C-terminal tail supports the uptake of the carotenoid and that structural changes (i.e., β5/β6 loop movement) assist to better host the carotenoid molecule within the first monomer as a moiety of an A-type CTDH dimer. Second, after a local energetic minimum is reached, reorganization of the monomers yields the F-type dimer while undergoing another structural change in the second monomer cavity to facilitate carotenoid binding.

### CTDH role and carotenoid transfer directionality

In a previous publication, we concluded that the role of CTDH is to help the assembly of holo-HCPs^25^. We proposed that the carotenoid transfer process could be unidirectional, i.e., from membranes to CTDH to HCPs. The fact that holo-AnaHCP4, -HCP3, or -HCP2 cannot transfer its carotenoid to the apo-AnaCTDH supported our hypothesis, suggesting it to be a possible in vivo scenario. Here it is shown that holo-AnaCTDH can supply the carotenoid to apo-HCP1 and apo-HCP4 with similar efficiency (Fig. 4b). In addition, holo-HCP1 is formed only in the presence of CTDH since HCP1 (like HCP4) is unable to extract the carotenoid from the membrane (at least in vitro) (Fig. 4d and Muzzopappa et al. (2017)^25^). Thus the presence of a CTDH is essential for the formation of all HCPs^25^.

Nevertheless, here we showed that, at least in vitro, HCP1 (but not HCP2, HCP3, and HCP4) can transfer its carotenoid to the apo-AnaCTDH. The question arises whether this process can occur in vivo as well. It is important to note that HCP1 was proposed to be a carotenoid carrier rather than a ^1^O_2_ or phosphate-buffered saline fluorescence quencher. Thus the fact that it can supply the carotenoid to the CTDHs supports this hypothesis. This result also suggests that the structure of HCP1, which is similar to the one of the NTD-OCP^19^, must be slightly different from that of other HCPs, at least concerning the interface with the CTDH, since it is the only HCP that is able to transfer the carotenoid to the various CTDH isoforms.

The results described here indicate that primary and tertiary structures of both CTDH (and CTD) and HCPs (and NTD) determine the possibility and/or the directionality of carotenoid transfer from one to the other. Slight structural modifications generated during evolution most probably appeared to suppress disadvantageous carotenoid transfer pathways and to add more effective and adaptive means of regulation.

### Structural position of holo-AnaCTDH subunits yields absorption differences compared to OCP^O^

When comparing the OCP^O^ structure and the F-type apo-AnaCTDH dimer, the NTD-equivalent, second CTDH monomer is positioned differently in space with respect to the first (CTD super-positioned) CTDH monomer, as can be seen in Supplementary Figure 12. While the carotenoid cavity in OCP^O^ is substantially tilted between the two domains, it is relatively straight in the apo-AnaCTDH dimer. Indeed, the carotenoid in OCP^O^ adopts a bent conformation, while it has a planar conformation in the holo-NTD variant^16^. The increased chromophore planarity and rotation of the terminal rings are known to induce the red shift of absorption peak due to elongation of the effective conjugation length^36,37^. While some structural alterations are anticipated upon carotenoid uptake by the apo-AnaCTDH, the overall axial positions of the two monomers are not expected to be dramatically changed. Thus the carotenoid cavity in the CTDH dimer will allow a planar conformation of the carotenoid. This should be clarified by structural studies using holo-CTD(H) dimer.

### CTDH carotenoid uptake and delivery cycle

In chloroplasts and cyanobacteria, various redox-activation processes of enzymes in the stroma exist. These activations occur via ferredoxin, ferredoxin-thioredoxin reductase, and thioredoxin (Trx)^38,39^. Trx can also be reduced by NADPH-Trx reductases. Trx also acts as an electron donor for antioxidant defense systems (e.g., peroxiredoxins, catalases)^40^. From the known functions of Trx, it can be clearly inferred that proteins with oxidized disulfide bonds exist in cyanobacterial cytoplasm under various conditions. The CTDH dimer therefore could also be a Trx-regulated protein (and/or other regulatory agent).

**Fig. 5:**
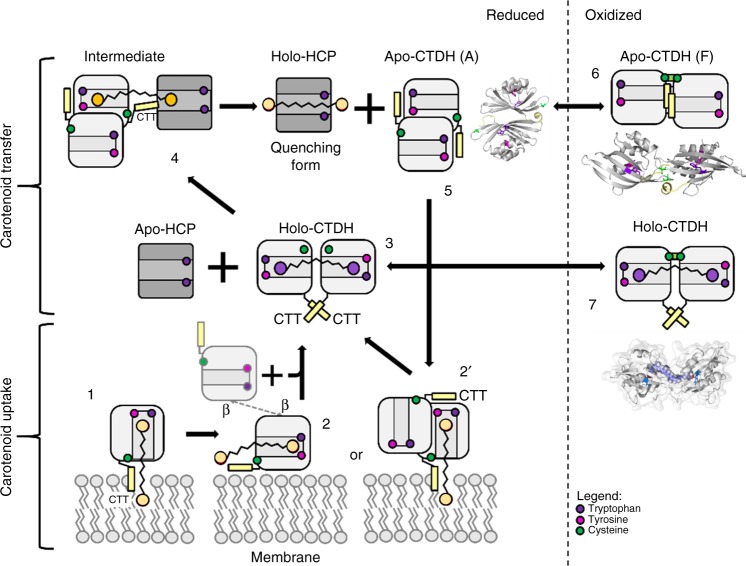
CTDH carotenoid uptake and delivery cycle. apo-AnaCTDH monomer (1) or a dimer in its A-type state (2′) may extract a carotenoid molecule from the membrane with the assistance of the C-terminal tail (labeled as CTT in the figure). Then a holo-dimer is formed (3) in an F-type state (with association of another monomer; 2) Then the holo-CTDH can either be redox regulated in its F-type state between the reduced (3) and oxidized states (7) or interact with apo-HCP to allow carotenoid transfer through the A-type state with the assistance of the C-terminal tail (4). This yields the holo-HCP and the apo-CTDH dimer that can undergo A-to-F type transition and either be in its reduced state (5) and thus can take another carotenoid molecule or be modified to its oxidized states (6). Different carotenoid molecule colors represent different binding modes and thus different spectroscopic characteristics

Based on these data and previous work of our laboratories, we can suggest a mechanism (Fig. 5) describing carotenoid delivery and uptake by a clade 2 CTDH. In vivo, in low light conditions or darkness (where no photoprotection is needed), Trx is more oxidized owing to low photosynthetic activity, and thus holo- and apo-CTDH dimers are stabilized by the disulfide bond between monomers that inhibits carotenoid uptake and delivery (Fig. 5, steps 6 and 7). Under conditions where photoprotection against oxidative stress is needed (reducing conditions, high light, low CO_2_), the Trx pool is largely reduced which in turn reduces both the apo- and holo-CTDH dimer to facilitate carotenoid transfer (Fig. 5, step 3) and uptake (Fig. 5, step 5). When reduced, and with the aid of HCP, the holo F-type dimer is converted to an A-type dimer, leaving the carotenoid bound to one monomer, bringing it one step closer to be transferred to the HCP. Then, by the assistance of the C-terminal tail, the carotenoid can be transferred directly to HCPs (Fig. 5, step 4).

After carotenoid transfer, the C-terminal tail remains near the carotenoid cavity and the CTDH can dimerize to yield the F-type apo-CTDH shown in this work. This apo-dimer is stable and will be able to receive a new carotenoid molecule only upon reduction of the disulfide bond. The uptake of the carotenoid from the membrane (and from HCP1) can be done by either a monomer (Fig. 5, step 1) or a reduced dimer (Fig. 5, step 2) in its A-type state; however, in either case the extension of the C-terminal tail toward the carotenoid site facilitates its translocation toward the designated cavity in the CTDH. A second monomer (Fig. 5, step 2′) is then assembled in order to co-encapsulate the carotenoid and form the F-type holo-CTDH (Fig. 5, step 3), which under regular conditions undergoes an oxidation event to yield the carotenoid-locked holo-CTDH (Fig. 5, step 7). We believe that F-type apo-dimer can be a result of two possible scenarios, which are not necessarily mutually exclusive. First, it is possible that carotenoid uptake is already completed during or immediately after CTDH synthesis before the formation of the dimer and disulfide bond. In this case, the F-type apo-dimer will be an intermediate stage along the CTDH carotenoid cycle. Nevertheless, it is also possible that the protein dimer scaffold can be formed prior to carotenoid uptake, and thus interchange between redox states can occur. In this case, F-type apo-dimer would be a preliminary stage of the cycle. These alternatives require further investigation.

## Methods

### Construction of plasmids containing *Synechocystis ctd-ocp* and *Anabaena ctdh* and *hcp* genes for expression in *E. coli*

The construction of the plasmid containing the *Synechocystis* 6803 CTD used for SAXS experiments was previously described^26^. The construction of plasmids containing *Anabaena hcp1*, *hcp2*, *hcp3*, and *hcp4* genes were previously described^22^ and of those containing the *Anabaena WT ctdh* and mutated *ctdh-C103F* genes (pCDFCTDHAna-4940Ctag and pCDF-CTDHAna-C103F, respectively) were previously described^25^. The C-terminal tail was deleted by mutagenesis using the plasmid pCDF-CTDHAna-C103F and the primers F-4940-ΔC-terminal tail- (5′-GATCATCCACACCCAAATTAAACACCACCACCACCACCACTAGTCTTG-3′) and R-4940-ΔC-terminal tail (5′-TTTAATTTGGGTGTGGATGATCTGTTTTTCTTGGTTAAGAG-3′) to create the pCDF-CTDHAna-C103F-ΔC-terminal tail. In all these CTDH plasmids, the sequence coding for the His-tag were in the 3′ terminus of the gene (C-terminus of the protein).

To obtain N-terminal His-tagged CTDHs, the primers F-4940-BamHI (5′-CACACGGATCCGAAAGCTGCTGAGTCTCTCCC-3′) and R-4940-NotI (5′-CATTATGCGGCCGCCTTATTGATGACAGCGCCCC-3′) were used to amplify the *ctdh* gene from genomic DNA of *Anabaena* PCC 7120 and cloned in pCDFDuet-1 digested by BamHI and NotI. The point mutation C103F (see Muzzopappa et al. (2017)^25^ for primers) and the deletion of the C-terminal tail (using F-4940-ΔC-terminal tail (5′-GATCATCCACACCCAAATTAAATAGTCTTGATTAATTACTTTAAC-3′) and R-4940-ΔC-terminal tail) were introduced by mutagenesis. Then a part of the N-terminal prolongation present in these plasmids was excised by mutagenesis using the primers F-4940-ΔBamHI (5′-CATCACCATCATCACCACAAAGCTGCTGAGTCTCTCCC-3′) and R-ΔDuet (5′-GTGGTGATGATGGTGATGCATGGTATATCTCCT-3′) to create the plasmids pCDF-NHISCTDHAna-C103F and pCDF-NHISCTDHAna-C103F-ΔC-terminal tail. The proteins obtained using these plasmids contained only six His after the first Met and then the CTDH primary sequence.

### HCPs and apo-CTDH production, isolation, and purification

The production and isolation of apo and holo-HCPs (Lopez-Igual et al.^22^) and apo- and holo-AnaCTDH were previously described^25^. For the apo-Ana-CTDH isolation used to crystallization, lysis buffer (40 mM Tris pH 8, 10% glycerol, 300 mM NaCl, 1 mM EDTA, 1 mM PMSF, 1 mM caproic acid, 1 mM benzamidic acid, 50 µg mL^−1^ DNAse) was then passed through a French Press machine. Following removal of membranes, the supernatant was further purified using a nickel affinity column (Ni-Probond resin, Invitrogen). Proteins were eluted with 250 mM imidazole and followed by dialysis with 40 mM Tris-HCl pH 8. To further disassemble AnaCTDH oligomers into smaller assemblies, the isolated protein was treated with 2 M urea and put for an overnight incubation prior to size exclusion chromatography.

### Sodium dodecyl sulfate (SDS)–PAGE and Native-PAGE

Crude apo-AnaCTDH sample was analyzed as previously described^25^. SDS–PAGE was performed on a 15% polyacrylamide/2 M Urea in TRIS/MES system^41^. Non-denaturing gel electrophoresis was done to examine the oligomeric state of the apo-AnaCTDH. Following purification, CTDH was loaded onto 15% native polyacrylamide gels (pH 8), and electrophoresis was conducted in a buffer at pH 8 (25 mM Tris-HCl, 192 mM glycine). Carbonic anhydrase (29 kDa) and α-Lactalbumin (14 kDa) were used as markers.

### Size exclusion chromatography

The crude apo-AnaCTDH oligomeric mixture was applied onto an analytical size exclusion chromatography (BioSEC-5, 1000 Å, Agilent, CA, USA) with 40 mM Tris-HCl (pH 8) used as elution buffer. To further purify AnaCTDH dimers, a 2 M urea-treated oligomeric mixture was applied onto a preparative size exclusion chromatography and eluted with a 40 mM Tris-HCl buffer (pH 8) containing 2 M urea. Ultraviolet detection was conducted at *λ*_abs_ = 225 nm with a flow rate of 0.5 mL min^−1^. Weight calibration was executed using the following markers: Thyroglobulin, bovine (669 kDa); Apoferritin, horse spleen (443 kDa); Albumin, bovine serum (66 kDa); Carbonic anhydrase, Erythrocyte, bovine (29 kDa), and Cytochrome C, horse heart (12.4 kDa).

### Crystallization and data collection

AnaCTDH oligomer crystals diffracting to 2.9 Å were obtained at 20 °C by hanging drop diffusion method when grown in 1.26 M NaH_2_PO_4_ and 0.14 M K_2_HPO_4_, pH 5 (being an optimized condition following INDEX HT screen, Hampton research). Large football-shaped crystals were obtained after several days and data sets were collected at Technion Center for Structural Biology (TCSB). AnaCTDH dimers in 2 M urea were crystallized at 15 °C by hanging drop vapor diffusion method (final urea concentration was 1 M). AnaCTDH crystals diffracting to a resolution of 2.43 Å were obtained by growth in 0.1 M citric acid, 25% w/v PEG 3350, pH 4.2 (optimized following INDEX HT screen, Hampton research). Smaller crystals were obtained after several days, and data sets were collected at the European Synchrotron Radiation Facility (ESRF) on beamline ID 30-A1 using MXPressE automatic data collection service. The data sets for both oligomeric states were scaled and merged using MOSFLM^42^ and SCALA^43^, respectively. Molecular replacement runs were carried out using Phaser^44^. While the AnaCTDH oligomer structure was failed to be solved using molecular replacement, the structure of the dimeric AnaCTDH was solved and then refined using both NCSref and Phenix.refine^45^. Structural solution inspections and manual modifications were made using Coot^46^. PDBREDO server^47^ was utilized to minimize errors prior to deposition to the PDB. The structure was then examined and compared to other structures using PyMoL^48^. A section of the composite Fo-Fc omit map calculated using the Phenix protocol and visualized in Pymol is shown in Supplementary Figure 6. The overall *B*-factors of the CTDH structure are higher in the peripheral surfaces of the protein, perhaps due to the presence of 1 M urea in the crystallization liquor (Table 1). Three urea molecules were modeled into densities too large to be modeled as solvent and not corresponding to bound ionic species. The *B*-factors of the urea molecules are higher than the average protein *B*-factors, further indicating that the urea molecules required to avoid oligomerization are weakly bound to the protein elements.

### Homology modeling

A homology-based model of the AnaCTDH structure was built using Swiss-model^49^, utilizing the CTD structure from OCP (PDB code: 5UI2), to serve as a template for MR attempts.

### Docking

Docking simulations were executed using Swissdock server^35^, with the structure of the monomer, dimer, and monomer/dimer without C-terminal tail as the rigid body and canthaxanthin as the ligand to fit.

### SAXS of apo-OCP-CTD

Apo-CTD from *Synechocystis* (residues 165–317), carrying an uncleavable N-terminal hexahistidine tag^26^, was analyzed in 20 mM Tris-HCl buffer (pH 7.6) containing 150 mM NaCl, 0.1 mM EDTA, 2 mM dithiothreitol, and 3% glycerol by synchrotron SAXS at the EMBL P12 beam line (PETRA III, DESY Hamburg, Germany)^50^. SAXS curves collected in a batch mode (1 s exposure time, collected as 20 × 50 ms frames) at different protein concentrations showed substantial concentration dependence, in line with the previous observations using size exclusion chromatography^26^. Since no extrapolation was possible, to ensure the predominance of the dimeric species, the SAXS data at the highest protein concentration (270 µM per monomer) were analyzed and used for modeling. The averaged SAXS curve for the sample was buffer subtracted in *PRIMUS*^51^. No radiation damage was detected by inspection of the time course of the scattering for protein frames. The Guinier region was linear and was used to determine experimental radius of gyration, *R*_g_. Pairwise distance distribution, *P*(*r*), was calculated by *GNOM*^52^ at *s* ≤ 0.28 Å^−1^ to determine the maximum particle dimension, *D*_max_, and the Porod volume. Ab initio shape reconstruction was performed using *DAMMIF*^31^ and three best-fitting models were averaged using *DAMAVER*^32^ to reveal the average core common among the models (*DAMFILT*). Theoretical SAXS curves and fitting to the experimental data were calculated using *CRYSOL*^53^. The core models of the apo-CTD dimers were built by superposition of the *Synechocystis* OCP-CTDs (PDB code: 4XB5; residues 173–305 out of 317) onto the crystallographic *Anabaena* apo-CTDH dimers in order to preserve the subunit interfaces. Few clashes in the amino acid side chains and flexible loops were relieved manually in *Coot*^46^ in the case of the F-type apo-CTD dimer preserving the head-to-head orientation. In the case of A-type apo-CTD dimer, the local protein–protein docking of the two subunits was required to account for local differences between apo-CTD and apo-CTDH sequences and to relieve clashes. The *RosettaDock* server^33^ was used with the default set of parameters, which resulted in a more connected and realistic top-scoring model (Supplementary Figure 7), devoid of steric clashes but preserving the back-to-back subunit orientation. On the basis of the fixed core dimers (either A or F) thus obtained, the unstructured parts of the protein (23 residues in the N-terminal tail and 12 residues in the C-terminal tail) were modeled using *CORAL*^54^ to minimize the difference between the model-derived and experimental SAXS curves. The modeling procedure was repeated ten times for each scenario to verify that a stable solution is found and the data corresponding to the best-fitting solution are presented along with the statistical analysis using χ^2^ and correlation map *P* value^34^. To assess the robustness of the solution, the hypothetical modeling by either apo-CTD monomer or trimer (supplemented with the flexible termini) was also performed but yielded much worse fits to the SAXS data.

### Absorbance measurements and experiments of carotenoid transfer

Absorbance spectra and kinetics of carotenoid transfer between CTDH and HCPs were measured in a Specord S600 spectrophotometer (Analytic Jena) at 23 °C. To study the carotenoid transfer from holo-proteins to apo-proteins, holo-HCPs to apo-CTDHs (1 holo-HCP per 2.5 apo-CTDHs molar ratio) and holo-CTDHs to apo-HCPs (1 holo-CTDH to 5 apo-HCP molar ratio) protein mixtures were incubated in 40 mM Tris-HCl buffer (pH 8) at 23 °C for 1 h in darkness. A triplicate of absorbance spectra were recorded for 1 h and carotenoid transfer was followed by changes in absorbance at 600 nm. To determine the percentage of carotenoid transferred, a spectral deconvolution was performed using Excel to fit the data to the sum of the reference spectra of the holo-proteins involved in the experiment (described in ref. ^26^). To study the carotenoid transfer from membranes to HCPs and CTDHs, 12 µM apo-dimers were incubated with an *E. coli* canthaxanthin-containing membrane suspension (48 µM canthaxanthin, measured by acetone extraction) at 33 °C for 1 h in darkness. Holo-protein formation was measured by absorbance spectroscopy after precipitation of membranes. The percentage of holo-protein formed was determined by comparing the spectra of 100% holo-proteins (at 12 µM) to those of the supernatant.

### Data availability

The data sets generated during the current study are available in the Protein Data Bank repository, [https://www.rcsb.org/] under accession code 6FEJ. All other data sets generated during and/or analyzed during the current study are available from the corresponding author on reasonable request.

## Electronic supplementary material

Supplementary Information
