# Supplementary material for: Structural rearrangements in the C-terminal domain homolog of Orange Carotenoid Protein are crucial for carotenoid transfer
Source: Commun Biol. 2018 Aug 27;1:125. doi: 10.1038/s42003-018-0132-5 (PMC6123778; doi:10.1038/s42003-018-0132-5)
Supplement: Supplementary file 1 — Supplementary Information [file 42003_2018_132_MOESM1_ESM.docx]

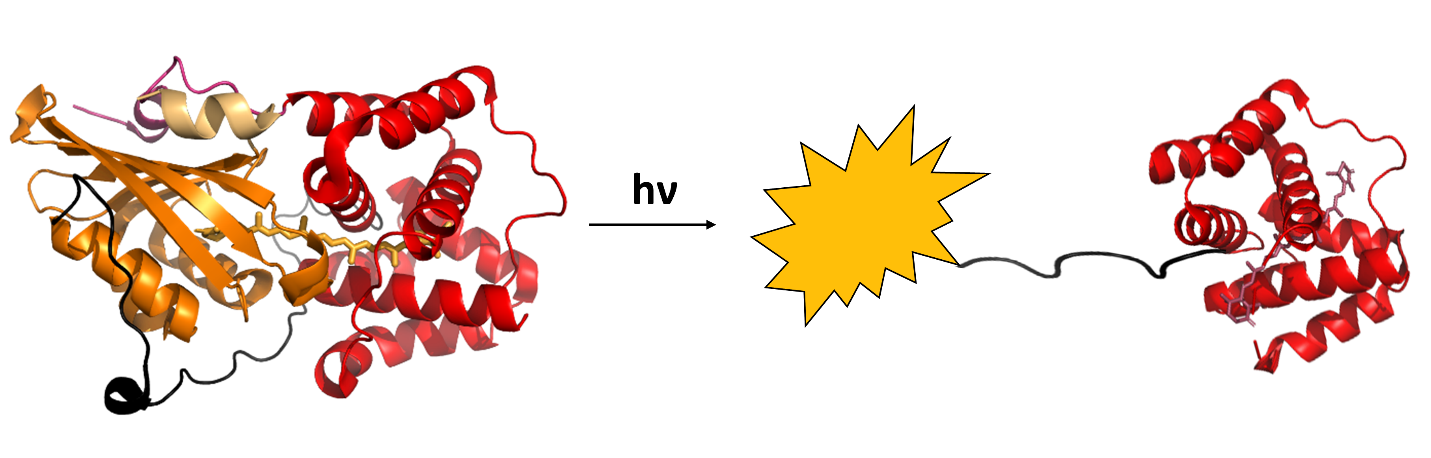


**Supplementary Figure 1. OCP^O^ to OCP^R^ photo-conversion induces major structural changes.** **(A)** The inactive OCP^O^ structure (PDB code: 5UI2). CTD (orange) and NTD (red) are connected through the flexible linker loop (black) and encapsulate a carotenoid molecule (3'-OH-Echinenone, yellow-orange). The CTD-C-terminal tail (pale-orange) is situated outside the interdomain interface. The N-terminal arm (NTE) (dark pink behind C-terminal tail) is found in interaction with the exterior side of the CTD. **(B)** upon high light irradiation, the OCP^O^ structure is disrupted to yield OCP^R^. Upon this transition, the carotenoid remains bound to the NTD and slides 12Å into a designated tunnel. The CTD (orange polygon – crystal structure unknown) and NTD are no longer in association and are held together only by the flexible loop (black) whose structure is also unknown. Three factors stabilize the OCP^O^ – the carotenoid, the interface between the domains (especially Arg155-Glu244 and Trp277-Asn104 salt and hydrogen bridges) and the interface between the NTE and an external face of the CTD.


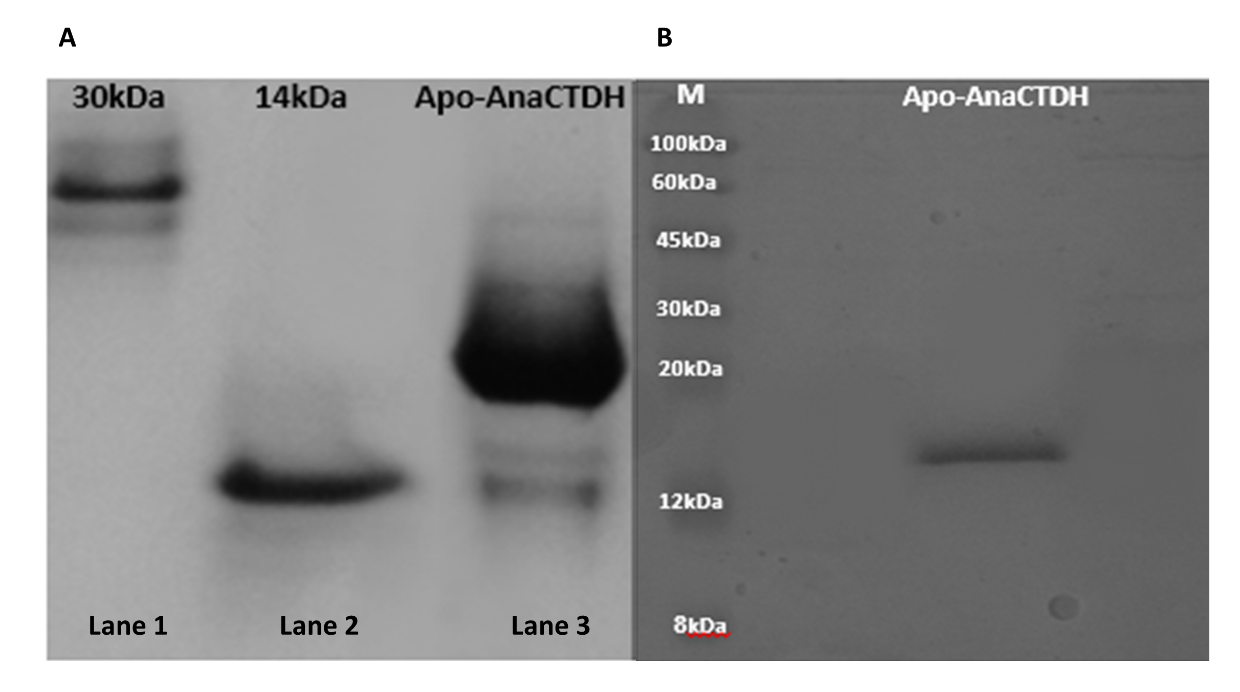


**Supplementary Figure 2. Assessment of the oligomeric state of apo-AnaCTDH by native-PAGE and SDS-PAGE.** (**A**) A typical run of an apo-AnaCTDH dimer in native-PAGE (lane 3) with markers (lanes 1, 2). (**B**) SDS-PAGE reveals that the reduced form of apo-AnaCTDH is in good accordance with a monomeric MW (15kDa).


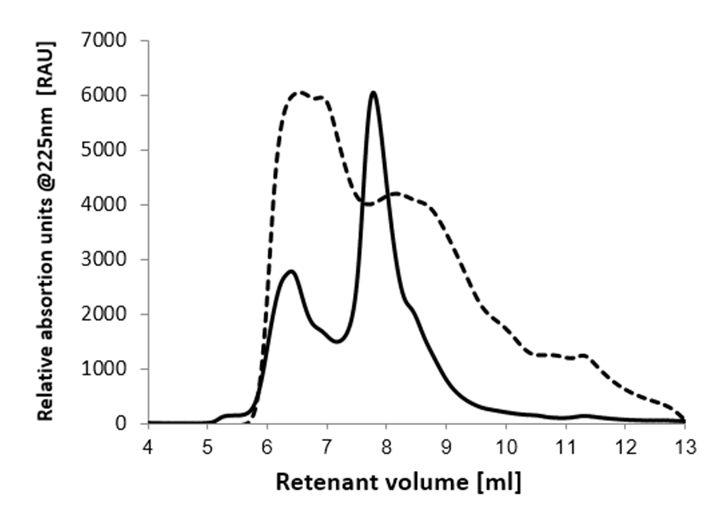


**Supplementary Figure 3. Effect of urea on the oligomeric state of CTDH observed by size exclusion chromatography.** Crude CTDH mixture elution profile (dashed bold line); following introduction of 2M urea (solid bold line), a partial shift into a smaller MW assembly is observed, matching the predicted mobility of an AnaCTDH dimer, by column calibration.


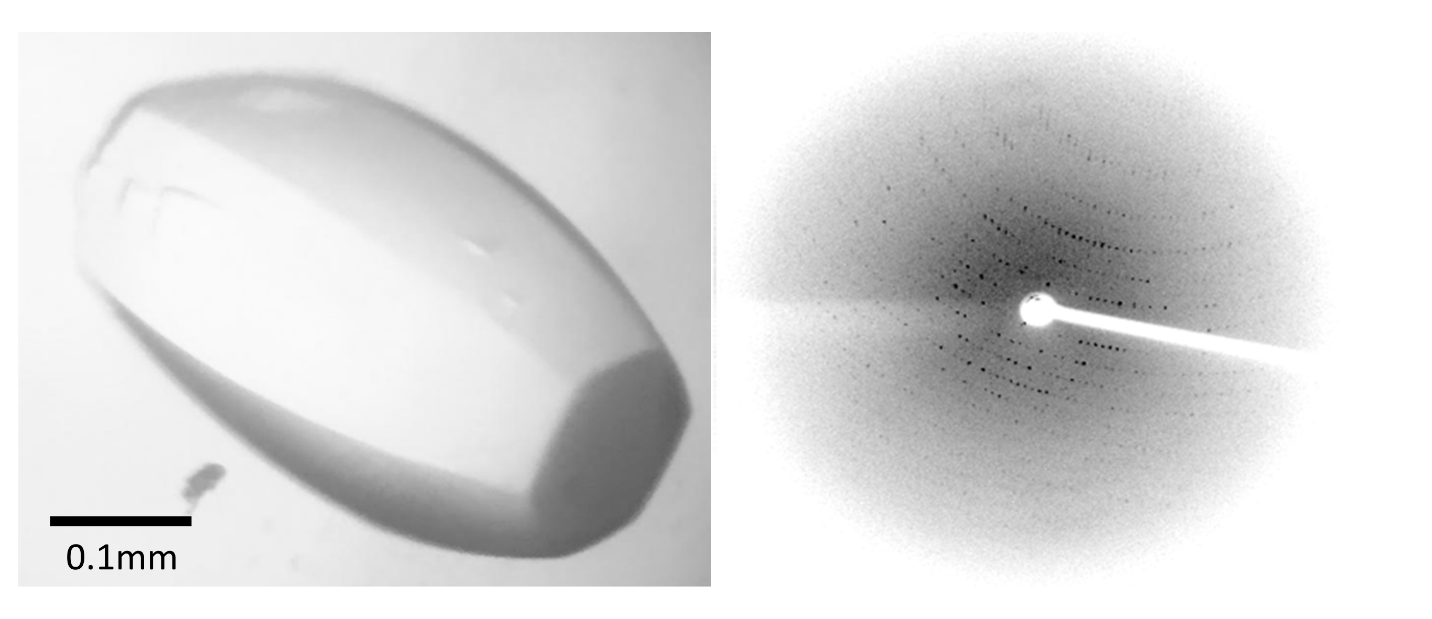

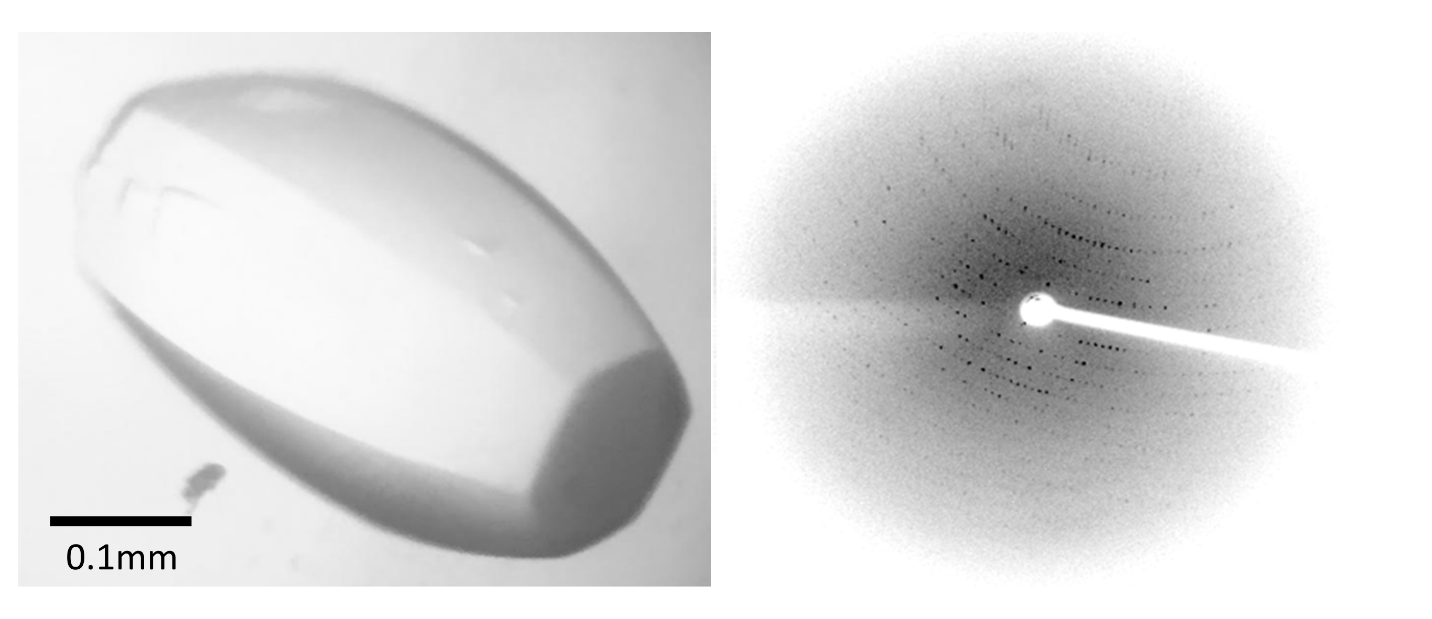


**Supplementary Figure 4. High molecular weight oligomer of CTDH crystalizes and diffracts, indicating the presence of a specific complex.** (A) Typical football shaped crystal generated from the native solution (scale bar = 0.1mm). (B) Diffraction pattern sample from crystal in panel A.


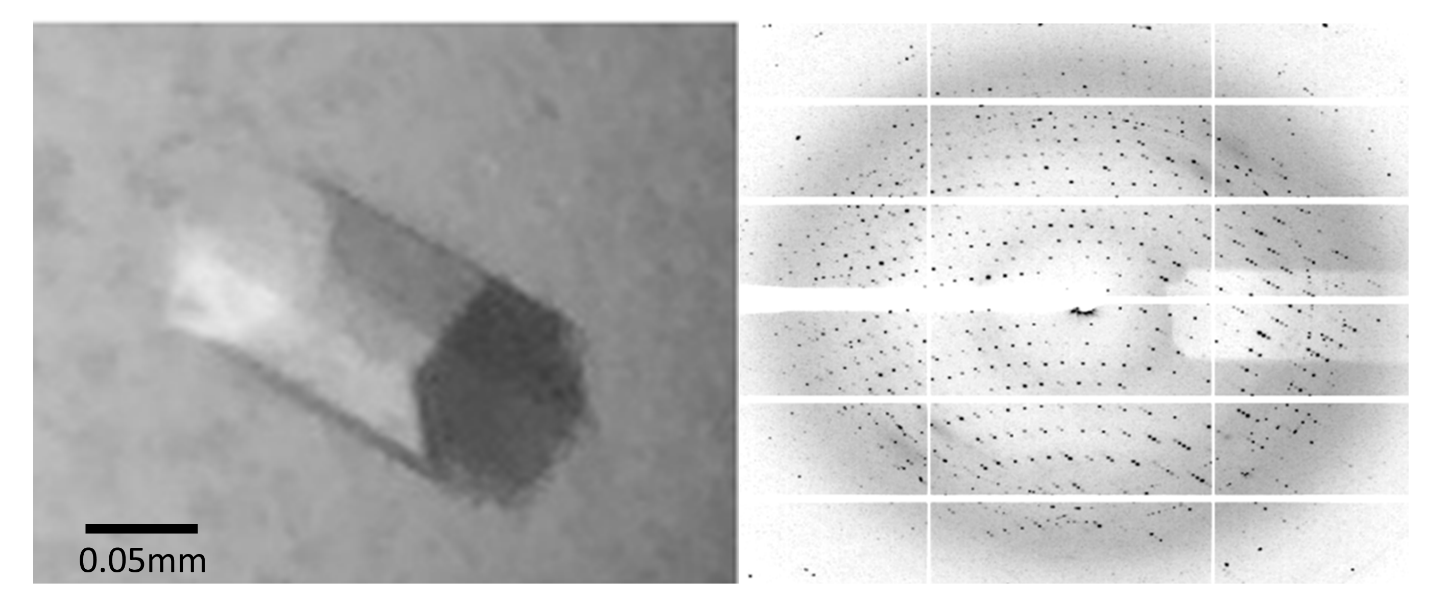

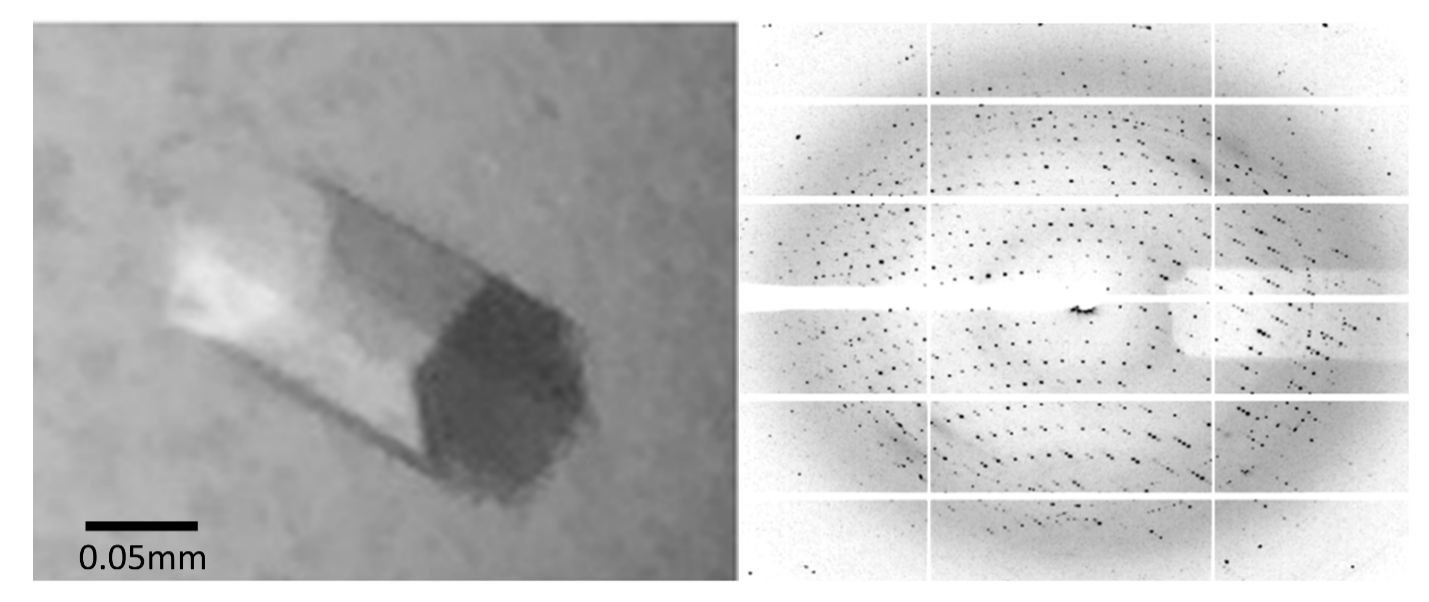


**Supplementary Figure 5. 2M urea treated CTDH crystalizes and diffracts.** (A) Typical hexagonal cylinder-shaped crystal generated from the 2M urea-treated CTDH solution (scale bar = 0.05mm). (B) Diffraction pattern sample from crystal in panel A.


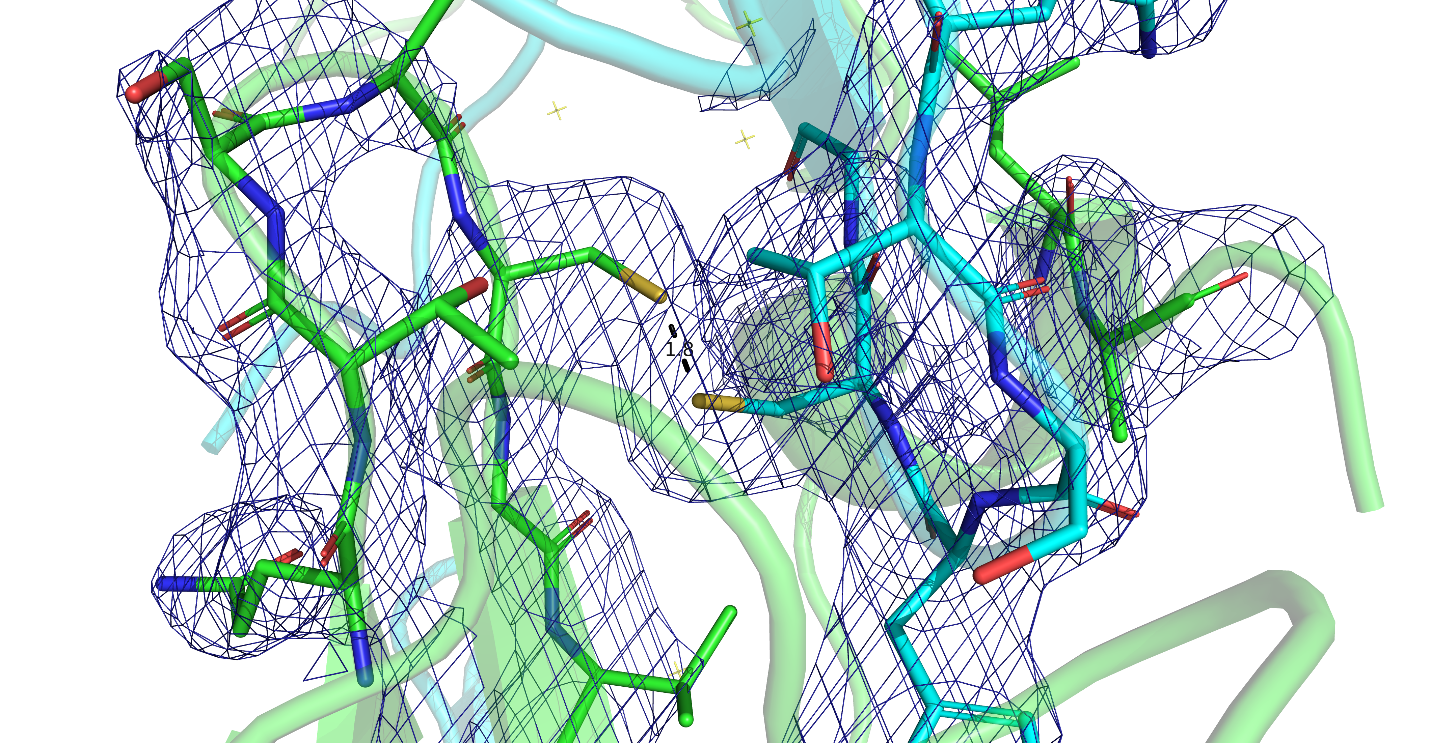


**Supplementary Figure 6.** Representative example of CTDH structure electron density. Composite Fo-Fc omit contoured at 1σ overlaid onto disulfide bond between of monomer A (green cartoon and carbons) and monomer B (cyan cartoon and carbons) in the adjacent asymmetric unit. The sulfur atoms are in yellow stick, and the disulfide bond is 1.8Å in length.


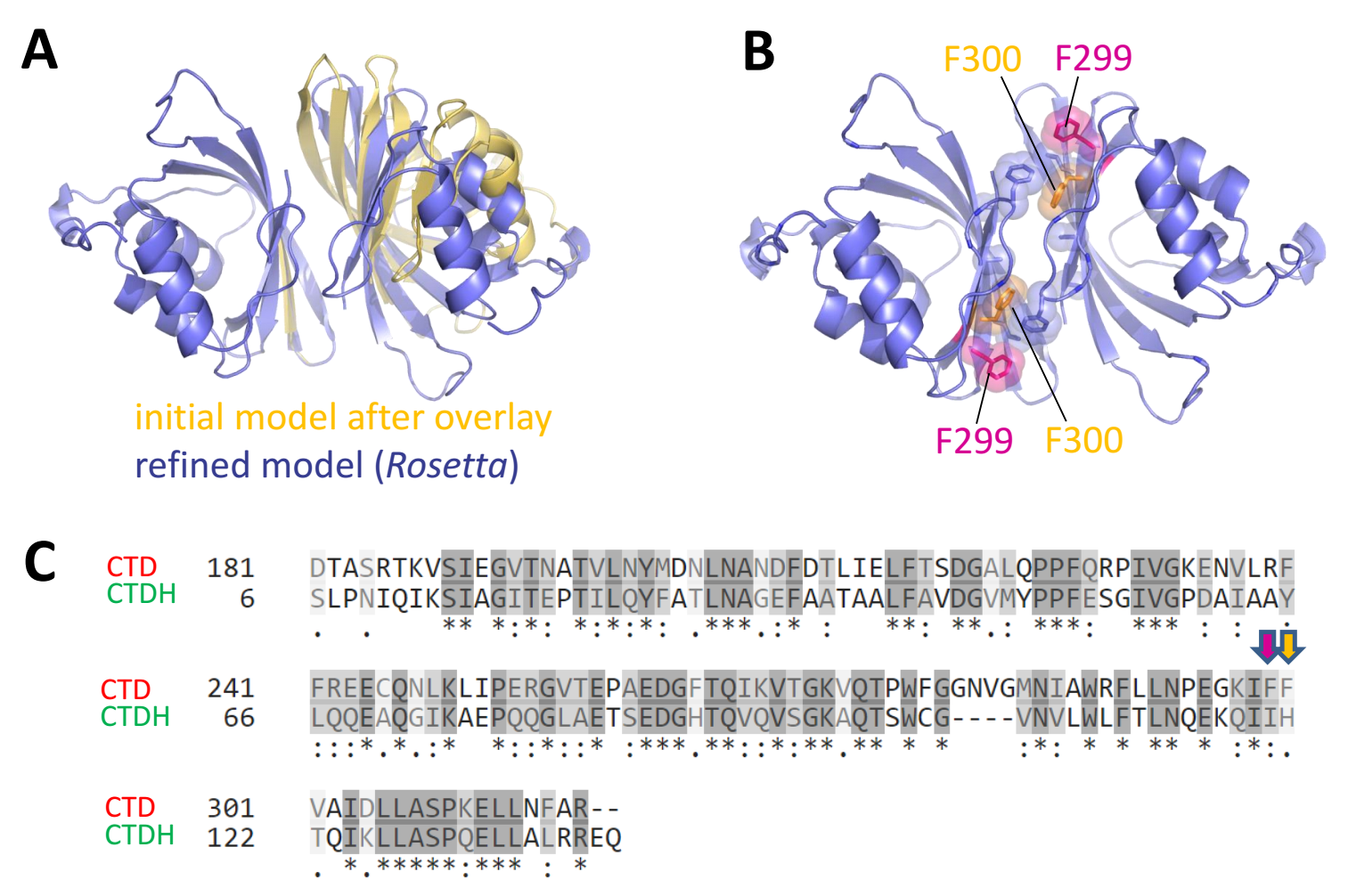


**Supplementary Figure 7. Modeling of the Apo-CTD dimer in a “back-to-back” conformation. (**A) The A-type of Apo-CTD dimer was obtained by a spatial overlay with the crystal structure of Apo-CTDH dimer (yellow) and then refined by using local protein-protein docking in *RosettaDock* (violet). (B) The refined model was devoid of steric clashes and revealed the potential role of the residues F299 and F300 (numbering of the full-length OCP from *Synechocystis*) in the formation of Apo-CTD dimer in contrast to Apo-CTDH, which lacks these phenylalanine residues. The absence of these residues in AnaCTDH may explain the difference in the ability of AnaCTDH and OCP-CTD to dimerize in the absence of carotenoid. (C) The alignment of primary structures of OCP-CTD and AnaCTDH showing sequence similarity (greyscale) and the difference in positions 299 and 300 of *Synechocystis* OCP-CTD. Note the 4 amino acid gap in AnaCTDH corresponding to the loop between β5 and β6 strands (see text for more details).


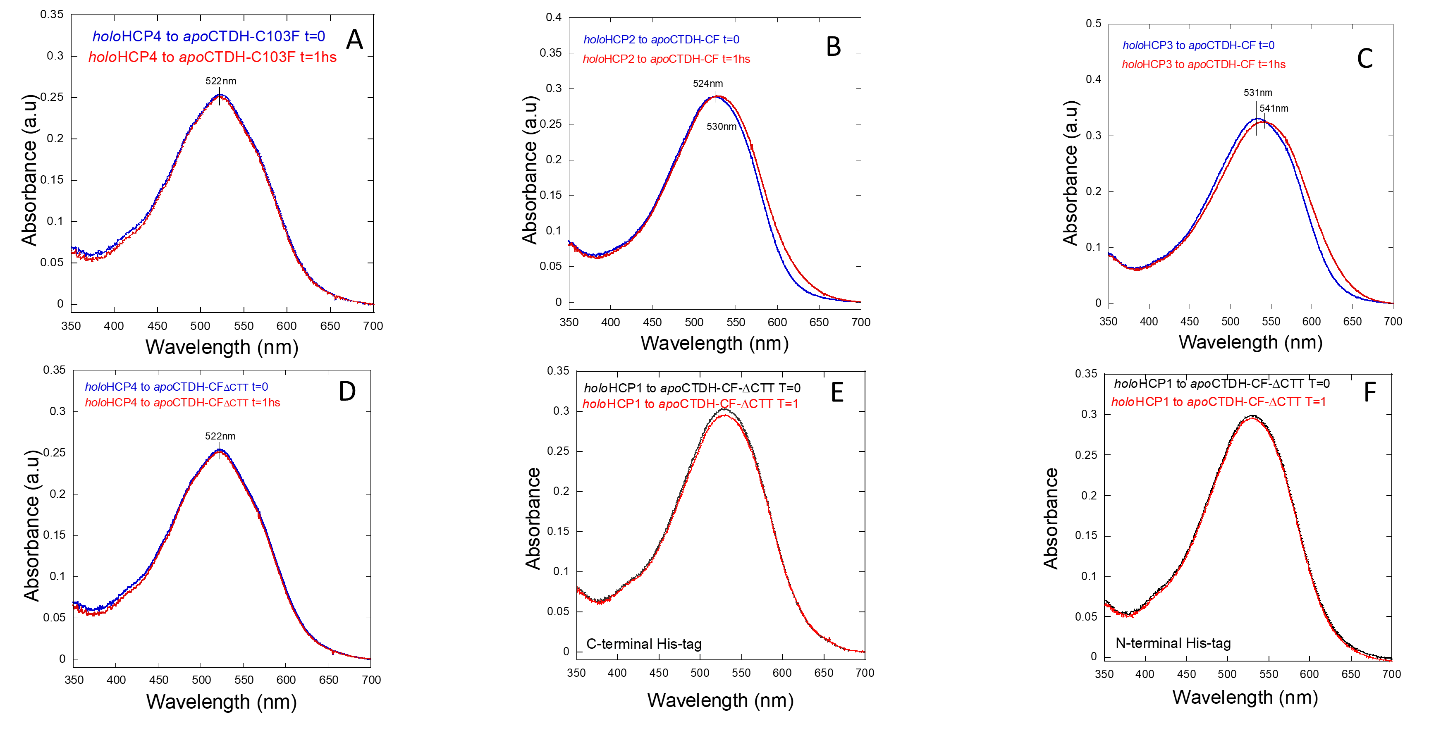


**Supplementary Figure 8. Carotenoid transfer from HCPs to Ana-CTDH-C103F (A-C) and Ana-CTDH-C103F-ΔC-terminal tail (D-F).** Absorbance spectra at time=0 and after a one hour incubation of (**A**) holo-HCP4 and apo-CTDH-C103F, (**B**) holo-HCP2 and apo-CTDH-C103F, (**C**) holo-HCP3 and apo-CTDH-C103F and (**D**) holo-HCP4 and apo-CTDH-C103F-ΔC-terminal tail-Cter, (**E**) holo-HCP1 and apo-CTDH-C103F-ΔC-terminal tail-Cter, (**F**) holo-HCP4 and apo-CTDH-C103F-ΔC-terminal tail-Nter. Holo-HCP4, HCP3 and HCP2 are not able to transfer the carotenoid to Ana-CTDH (A to C). holo-HCP4 and HCP1 are also unable to give the carotenoid to apoCTDH-C103F-ΔC-terminal tail (D-F). CTDHs used in these experiments shown in A to E have the His-tag in the C-terminus. The same results were obtained using the CTDHs containing the His-tag in the N-terminus.


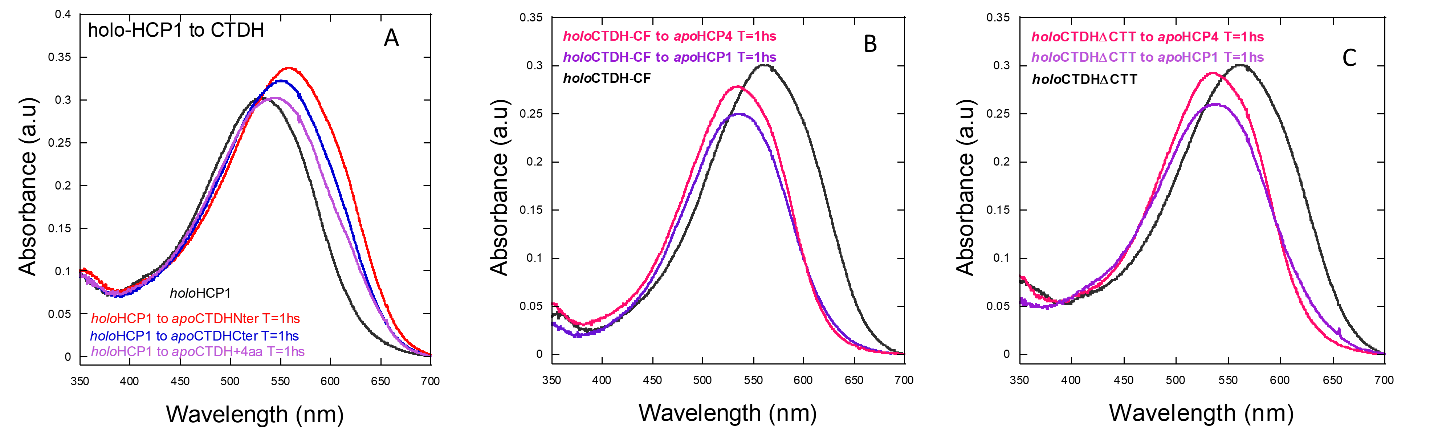


**Supplementary Figure 9. Carotenoid transfer from holo-HCP1 to apo-CTDH-C103Fand apo-CTDH-C103F+4aa (A) and carotenoid transfer from CTDH-C103F (B) and holo-CTDH-C103F-ΔC-terminal tail (C) to apoHCP1 (violet) and HCP4 (fucsia)**. (A) Absorbance spectra at time=0 (black) and after a one hour incubation of holo-HCP1 with apo-CTDH-C103F-Cter (blue), apo-CTDH-C103F-Nter (red) and apo-CTDH-C103F+4aa (violet). (B and C) Initial and final absorbance spectra after one hour incubation of holo-CTDH-C103F (B) and holo-CTDH-C103F-ΔC-terminal tail (C) with apo-HCP4 (fucsia) and apo-HCP1 (violet).


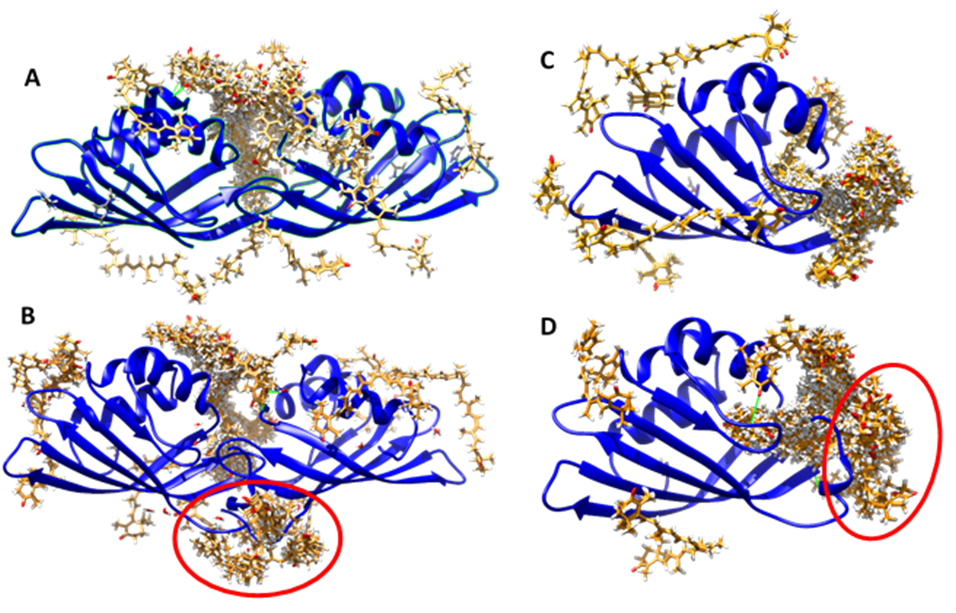


**Supplementary Figure 10. Docking of canthaxanthin chromophore to apo-AnaCTDH monomers and dimers (with/out C-terminal tail).** (**A**) Cartoon representation of CTDH dimer without C-terminal tail with all the carotenoid docking clusters super-positioned onto the protein (**B**) Same as A, but with C-terminal tail. (**C**) same as A, but with a monomer. (**D**) same as C, but with C-terminal tail. Circles denote the putative carotenoid binding site entrance.


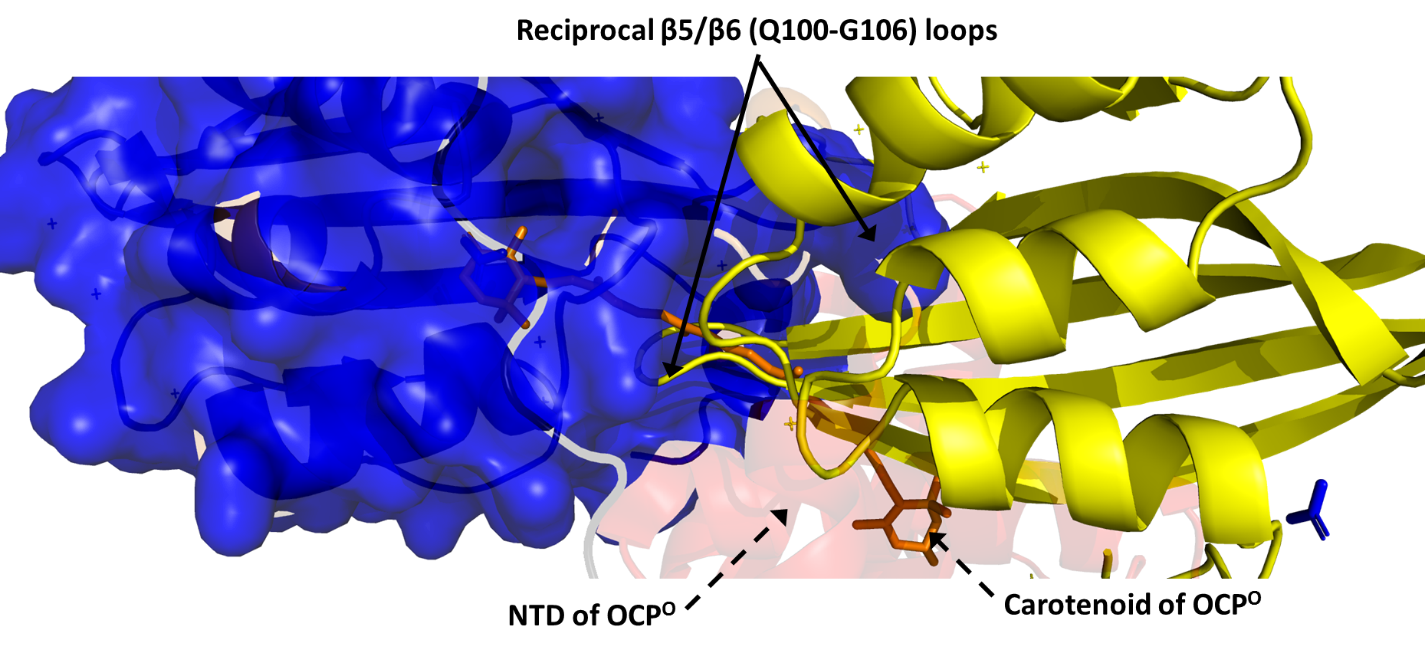


**Supplementary Figure 11. β5/β6 loops (Q100-G106) occupy carotenoid designated cavities in apo-AnaCTDH dimer.** CTDH monomer surface (blue) super-positioned with CTD of OCP^O^. NTD of OCP^o^ (transparent cartoon in the back, indicated using a dashed arrow) shares carotenoid (orange sticks, also indicated using a dashed arrow) with the CTD of OCP^O^. Second CTDH monomer (yellow cartoon) forms a homodimer in a different orientation with respect to NTD. Reciprocal Q100-G106 (QTSWCG) loops blocks the cavity where carotenoid should be situated in the holo form, as indicated by black full arrows (one points to the yellow loop penetrating the blue surface, and the second points to the second loop’ surface in blue, penetrating the yellow cartoon).


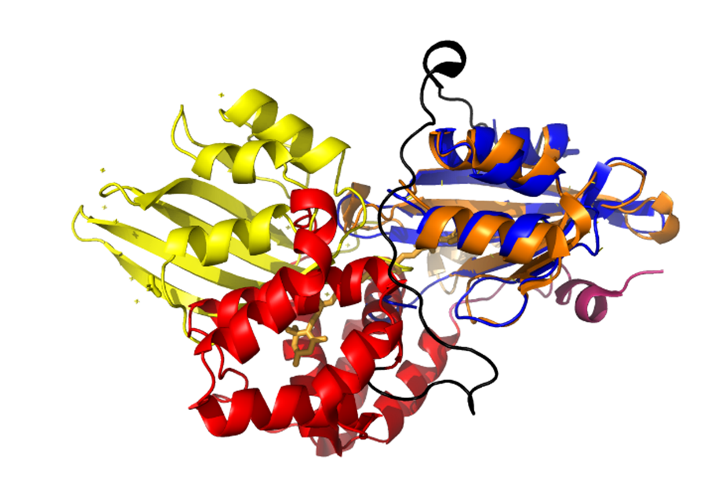


**Supplementary Figure 12. CTDH dimer planarity versus OCP^O^ bend.** Apo-AnaCTDH dimer is superimposed with OCP^O^ by overlapping an apo-AnaCTDH monomer (blue cartoon) with CTD (orange cartoon). NTD (red) facilitates bent carotenoid encapsulation (bright orange sticks) and connected to CTD through the flexible loop (black). The second apo-AnaCTDH monomer (yellow) is nearly on the same axis (horizontal) to the first monomer.
